# Grappling with tradition: the experiences of cisgender, heterosexual mothers and fathers in elective co-parenting arrangements

**DOI:** 10.1080/13229400.2023.2209060

**Published:** 2023-05-18

**Authors:** Susie Bower-Brown, Sarah Foley, Vasanti Jadva, Susan Golombok

**Affiliations:** a Centre for Family Research, University of Cambridge, Cambridge, UK; b Thomas Coram Research Unit, University College London, London, UK; c Moray House School of Education and Sport, University of Edinburgh, Edinburgh, UK; d London Institute for Women’s Health, University College London, London, UK

**Keywords:** Co-parenting, motherhood, fatherhood, gender, family display, qualitative

## Abstract

Elective co-parenting families, meaning two (or more parents) who are not in a romantic relationship having a child together, are becoming more common amongst cisgender, heterosexual parents. The study of elective co-parenting families offers researchers a unique opportunity to decouple co-parenting relationships from romantic relationships, but little research to date has explored their experiences. This study explored two research questions: why do individuals decide to enter into elective co-parenting arrangements? And how do they manage their co-parenting arrangement and their relationship with their co-parent? Interview data from 10 elective co-parents (5 mothers and 5 fathers) were analyzed according to the principles of reflexive thematic analysis. Sociological theorisations of family practices, family display and family thinking were utilized to make sense of the data. The results centred around two organizing themes (‘Reproducing the traditional family’ and ‘Modernising the traditional family’), and participants experienced a tension between these two ideas. Participants aimed to manage their co-parenting relationship with shared values and friendship, but defining their relationship was complex and gendered parenting patterns were ubiquitous. Findings add nuance to theorisations of family life and demonstrate that traditional parenthood ideologies remain pervasive, as parents aim to imagine and pursue parenthood on their own terms.

## Introduction

Elective co-parenting, meaning two (or more) parents who are not in a romantic relationship having a child together, has had a long history within the LGBTQ + community (Dempsey, 2012). However, recent media attention has been focussed on a new group of elective co-parents: cisgender, heterosexual (cishet) individuals who decide to have a child outside the context of a romantic relationship. The number of elective co-parenting arrangements is thought to be rising (Linton, 2020), but little is known about the experiences of these parents, with the small body of existing research focussing predominantly on LGBTQ + parents.

This article focuses on the experiences of cishet men and women who are raising children in the context of elective co-parenting arrangements.^1^ Drawing upon international qualitative interview data with 10 co-parents (5 mothers and 5 fathers) from 8 different families, we ask two key questions: why do individuals decide to enter into elective co-parenting arrangements? And how do they manage their co-parenting arrangement and their relationship with their co-parent?

We will now review the small body of literature on elective co-parenting, before discussing the research on co-parenting post-separation/divorce. We will then discuss the societal context in which elective co-parents decide to enter into such arrangements, including the differing representations and expectations of mothers and fathers, before discussing sociological theorisations on family life.

### Elective co-parenting

Elective co-parenting has received little academic attention, perhaps due to the ‘invisibility’ of these families, in that they resemble families that are co-parenting post-divorce/separation (Segal-Engelchin et al., 2005). Existing research on co-parenting has focussed on parental motivations for choosing elective co-parenting, finding that a key motivation for choosing co-parenting over other routes to parenthood (such as solo parenthood) is so that the child will have both a mother and father who are biologically related to the child (Erera & Segal-Engelchin, 2014; Herbrand, 2018a, 2018b; Jadva et al., 2015; Ravelingien et al., 2016). Much of this research focuses on the experiences of LGBTQ + parents. Whilst some research with queer and trans parents has found that co-parenting/polyamorous-parenting arrangements can enable resistance of traditional parenthood expectations (Bower-Brown, 2022; Pain, 2019; Vaccaro, 2010), other research with cis LGB parents highlights that many co-parents wish to create ‘traditional families’ (Herbrand, 2018a). For instance, parents have been found to form families which privilege biogenetic parenthood and follow traditional gender norms (e.g. with mothers as primary caregivers) (Erera & Segal-Engelchin, 2014; Gahan, 2019; Herbrand, 2018a). Herbrand (2018b) explored the way in which lesbian/gay co-parents in Belgium negotiated their parenting roles and responsibilities, finding that gender norms restricted the extent to which parents could create flexible and reflexive co-parenting arrangements. Moreover, research with gay male couples who co-parent with lesbian couples has found that these arrangements exhibit gendered power dynamics, with some fathers relying on a discourse of maternal responsibility compared to paternal choice, and some mothers exercising power and control over the father’s relationship with his child (Dempsey, 2012). In a study of 102 individuals (61 men and 41 women) who sought a co-parent online, 26% of heterosexual men reported that they would like to see their child every day, compared to 94% of heterosexual women (Jadva et al., 2015), suggesting that elective co-parenting arrangements may follow gendered patterns.

Research on families generally, and on co-parenting specifically, suggests differences between the way in which cishet individuals and LGBTQ+ individuals approach both being and becoming a parent (Bower-Brown, 2022; Jadva et al., 2015; Segal-Engelchin et al., 2012). Lesbian and bisexual women are more likely to identify co-parenting as an ‘ideal situation for bringing up a child’ than heterosexual women (Jadva et al., 2015). This suggests that cishet people, in particular, may view co-parenting as a ‘second-best’ alternative to parenting in the context of a romantic relationship (Segal-Engelchin et al., 2012). Heterosexual parents may also have different considerations when deciding who to co-parent with. For instance, heterosexual women who have pursued parenthood with gay men report avoiding heterosexual men due to a suspicion of their motives and to prevent romantic or sexual tension (Segal-Engelchin et al., 2012). This highlights the importance of researching the experiences of cishet men and women in co-parenting arrangements, as they may have unique experiences.

### Co-parenting post-separation

Generally, co-parenting refers to the collaboration in childrearing of two parental figures who share responsibilities for at least one child (Feinberg, 2003), and is a label most often applied to parents who have experienced divorce or separation. Historically, there has been much concern about the negative impact of divorce/separation on children (Lansford, 2009) and better child outcomes are associated with post-separation co-parenting that is typified by agreement over childrearing, satisfaction with the division of labour, support of the other parent’s role, and the joint management of family interactions (Feinberg, 2003; Lamela et al., 2016). Co-parenting between elective co-parents may be easier and less conflictual, given that they have not experienced separation (Segal-Engelchin et al., 2005), and thus studying these arrangements may shed further light on effective co-parenting practices.

Qualitative research on co-parenting post-separation has found that, whilst parents do renegotiate family practices after relationship breakdown, this renegotiation tends to follow gendered patterns (Solsona et al., 2020). Studies have focussed on the impact of ‘maternal gatekeeping’, defined as a mother’s desire to control parental decision making, on family processes (Schoppe-Sullivan & Fagan, 2020), with higher levels of maternal gatekeeping being associated with lower levels of paternal involvement (Fagan & Barnett, 2003). Additionally, gendered pre-separation co-parenting practices have been found to be associated with gendered post-separation co-parenting practices (Solsona et al., 2020). Thus, elective co-parenting families offer the opportunity to explore co-parenting practices without the complication of separation and divorce.

### Motherhood, fatherhood and the ‘traditional family’

The ‘traditional family’ is generally thought to be composed of a cisgender, heterosexual mother and father who are living with their biogenetically related children. This family is also typically white, middle-class and married. Within this family form, mothers and fathers take on different responsibilities: the mother is responsible for the ‘private’ sphere (e.g. the home) and the father is responsible for the public sphere (e.g. paid work) (Coltrane, 2004). Whilst historical analysis has questioned the widespread existence of the ‘traditional family’ beyond the 1950s (Coontz, 1992), this family form remains an ideal to which other family types are compared to.

Contemporary expectations of mothers and fathers remain different to this day (Pedersen, 2012). Although conceptualisations of motherhood have changed over time, much academic research has focussed on the ongoing prevalence of ‘intensive mothering’ ideology, which outlines a motherhood that is emotionally, financially and labour intensive (Hays, 1996), and central to the identity of cis women (Faircloth, 2014). Despite being historically and culturally situated, ideologies around intensive mothering are prevalent worldwide, such that many parents across the world may view intensive mothering as the ‘proper’ way to parent (Faircloth & Gürtin, 2017). Intensive mothering ideology has been found to be widespread amongst both mothers and fathers (Pedersen, 2012), and mothers parenting post-separation have found that being a ‘part-time parent’ can be challenging to ideologies of intensive motherhood (Markham & Coleman, 2020).

Representations of fatherhood have shifted substantially over time, from the traditional model of fatherhood, which focussed on discipline, morality and breadwinning to that of the ‘new’ or ‘involved’ father, which focusses on the father’s role as an involved, sensitive caregiver (Freeman, 2003, Dermott, 2008). It has been noted that shifts in conceptualisations of fatherhood have not necessarily been reflected by changes in practice (Zadeh et al., 2022), with traditional models of fatherhood remaining common. This is evidenced in the moral panic around ‘fatherless families’ (Freeman, 2003; Pleck, 2010b), which is prevalent in the political sphere, with ex-UK Prime Minister David Cameron suggesting that the 2011 London riots were committed by ‘children without fathers’ (Cameron, 2011).

Discussions of motherhood/fatherhood ideology are relevant to elective co-parenting families in a number of ways. Co-parenting may offer mothers the opportunity to avoid intensive mothering ideology, in allowing them to share the burden of parenting with other highly involved parents (Bower-Brown, 2022). However, parenting outside of the context of a romantic relationship may also strengthen the ideals of intensive motherhood. For couples who parent together, it has been highlighted that there are competing discourses around ideal relationships (which should be committed to equal division of labour) and ideal parenting (which should involve ‘intensive parenting’, with more contribution from mothers) (Faircloth, 2021). Such competing discourses are absent within the co-parenting relationship, suggesting that principles of intensive mothering may be particularly prevalent in elective co-parenting families. Given the contrasting representations of traditional/new fatherhood, elective co-parenting families can also offer us the opportunity to understand fatherhood amongst individuals who are motivated to pursue parenting outside of a romantic relationship.

### Family practices and family display

When discussing representations of motherhood and fatherhood, it is useful to consider the sociological theorisations of family life. Morgan’s (2011) theorisations on family practices have been influential in redefining family as something that is done, rather than something that simply is. Family practice refers to the activities that family members do in relation to each other, and the way in which these practices define and reproduce family relationships. Finch (2007) further suggested the idea of family display, which refers to the notion that families do not simply ‘do family practices’, but that families must display themselves as a family, essentially illustrating to themselves and others that ‘this is my family and it works’ (Finch, 2007, p. 70). Finch (2007, p. 71) also notes that, ‘the need for display is greater as relationships move further away from those which are readily recognizable as constituting family relationships’. Elective co-parenting families may therefore have a unique relationship with family display, as they may be recognized as a traditional family or a family with separated parents, rather than an elective co-parenting family, and, as such, it is important to understand the way in which co-parents engage in family display.

Other researchers have offered extensions and critiques of these theoretical frameworks. Zadeh et al. (2022) suggested the notion of ‘documenting family’, which describes the importance of paper-work and official documentation to non-normative families, within contexts that delegitimise or exclude them. Indeed, family display is bounded by situational context and related to power, in that family displays exist within a cultural framework of what a ‘good’ family is (Heaphy, 2011). Nordqvist (2017) suggests that the concept of family practices is insufficient in capturing the ideological aspects of family life. Outlining the process of ‘genetic thinking’, which can more broadly be applied to ‘family thinking’, Nordqvist (2017) calls for researchers to more fully engage with the relationship between family practice and family discourse.

In the current study, we utilize these theoretical frameworks to understand the motivations and experiences of elective co-parents. In doing so, we aim to increase understanding of the way in which parents (who are not romantically involved) see, define, and manage their relationships with each other and their child, and, more generally, shed light on the role of the romantic relationship in expectations and experiences within parenting partnerships.

## Methods

### Sample

Participants were recruited through parenting connection websites and mailing lists (e.g. Pride Angel, Modamily, Pollentree), social media and snowball sampling. Parents were eligible to participate in the study if they had a child aged 0–12 within a co-parenting arrangement and defined themselves as raising the child jointly or with involvement of the child’s other biological parent. Parents interested in the project emailed the research team, and they were then provided with detailed information about the study. The current study reports on 10 cisgender, heterosexual co-parents from 8 families. This is a subsample drawn from a larger study; all other parents in the study were LGBTQ+ and were excluded from the current analysis in order to focus solely on the experiences of cishet co-parents. Of the 10 cishet co-parents, five were mothers and five were fathers; five met online using co-parenting websites and five entered the arrangement with someone they already knew. Participants were aged between 35 and 48 years old (*M* = 40.22 years old, *SD* = 4.27) living across the United Kingdom, Europe and North America. Mothers were aged between 36 and 47 years old (*M* = 42 years old, *SD* = 4.47) and fathers between 30 and 48 years old (*M* = 38 years old, *SD* = 6.7). Parents were on average well educated (70% undergraduate degree or higher), though income level varied from less than £10,000 to £75,000. The majority of the sample had never married (70%), and the majority of the sample had one child (*M* = 3.68 years old, *SD* = 2.05, range 6 months – 7 years old; 4 boys and 6 girls).

### Interviews

Written informed consent to take part in the study was obtained from all participants. The study received ethical approval from the University of Cambridge Psychology Research Ethics Committee. Each parent took part in a qualitative, semi-structured interview conducted by one of the research team in the family home or online via video conferencing software. The interview asked questions relating to the participants’ route to parenthood, including their reasons for choosing co-parenting, experiences of finding a co-parent and experiences of conception, pregnancy and birth. Questions also focused on their experience of being a parent, including their relationships with their child and co-parent, their thoughts more generally about co-parenting and their experiences within wider society. All interviews were audio-recorded and transcribed verbatim. In the results section, data are presented verbatim, although pseudonyms have been assigned to participants, identifying details have been removed, and some repeated words and filler words (such as ‘like’, ‘kind of’) have been removed to aid legibility. Further, ellipses indicate the omission of speech and square brackets indicate the modification of speech.

### Analysis

We analysed the data according to the principles of reflexive thematic analysis (TA), a theoretically flexible qualitative analytic approach which aims to identify, develop and interpret patterned meanings within a dataset (Braun & Clarke, 2021). We took an inductive approach to reflexive TA, due to the lack of existing empirical and theoretical engagement with elective co-parenting. This research took a critical realist approach, which involves both ontological realism (i.e. there is an objective reality) and epistemological relativism (i.e. our subjective realities are socially constructed) (Willig, 2016). Under this approach, reflexive TA can be utilised to explore the influence of social structures and hegemonic representations on individuals’ subjective realities and meaning making processes (Braun & Clarke, 2021). In this study, reflexive TA was utilised to explore the way in which cishet elective co-parents’ motivations and experiences were structured by dominant representations of motherhood, fatherhood and the ‘traditional family’.

The first stage of reflexive thematic analysis involves familiarization with the data, and all interviews were read twice by the primary author (SBB), who then engaged in close coding of the interview data. Once having coded all manuscripts, the codes were grouped according to conceptual similarity and these groups were developed into preliminary themes, which were discussed between authors (SBB and SF). Following this discussion, we identified two overarching themes relating to tradition. After having explored relevant empirical and theoretical literatures, and with the idea of ‘tradition’ in mind, the themes were again revised and developed. Illustrative quotations were identified, and the results were written up.

Reflexive TA holds researcher subjectivity and reflexivity to be central (Braun & Clarke, 2021) and the authors reflected on their positionality and assumptions throughout the data collection and analysis process. The analysis and interviews were largely conducted by the primary and secondary author respectively. The primary author is a queer, non-parent, who has conducted research on LGBTQ+ families. As such, the primary author generally occupied an outsider status to the participants, and may have been particularly attuned to instances in which parents undertook cisheternormative gender roles. The team of authors are comprised of both LGBTQ+ and non-LGBTQ+ individuals, and parents and non-parents, and all researchers are experienced in researching diverse families. As a team, the authors therefore occupied both insider and outsider statuses in relation to the participants (Dwyer & Buckle, 2009), and it is recognised that researchers with different positionalities may have interpreted the data in a different way.

## Results

**Figure 1.**
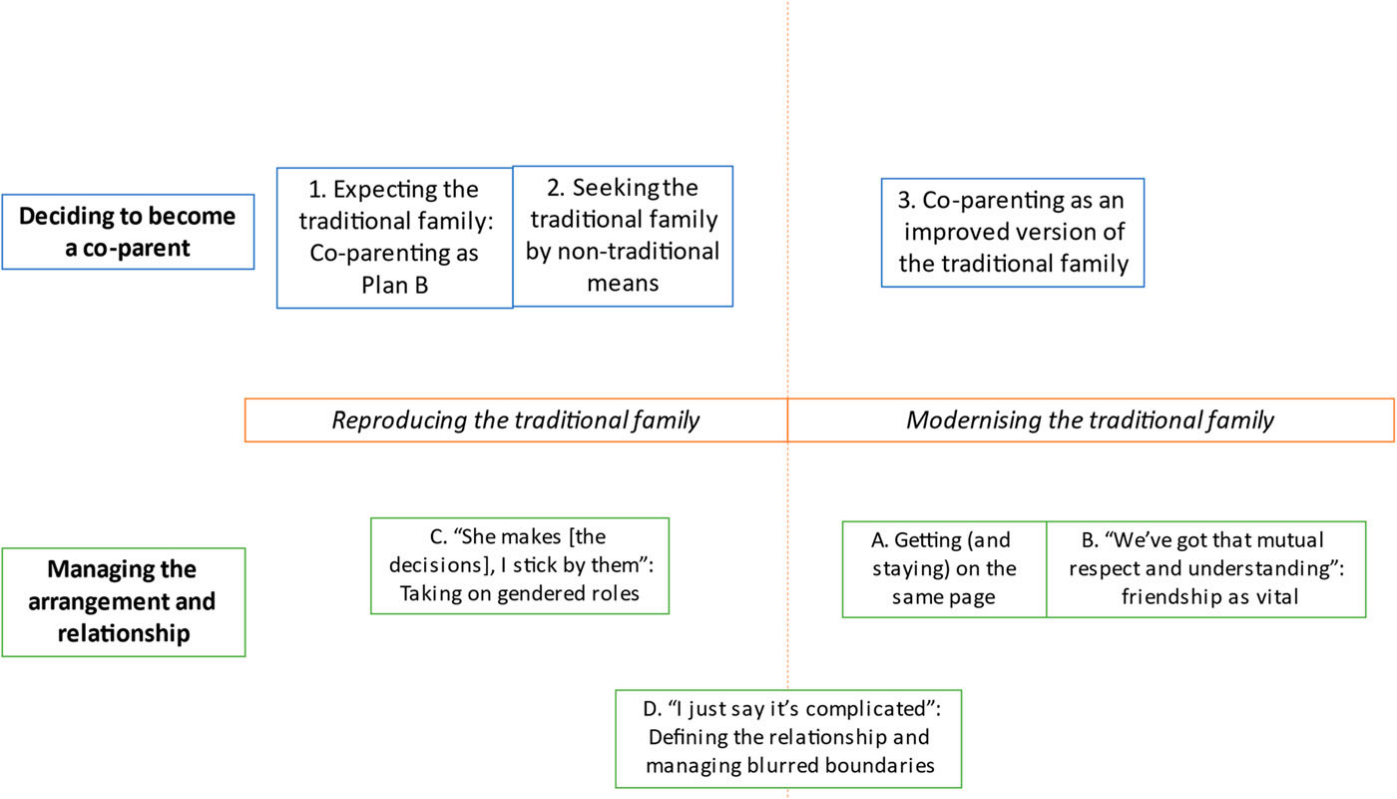
Thematic map depicting the research questions (in bold), the overarching themes (in italics) and the themes identified within the data.

This study had two research questions, firstly, why do people decide to enter into elective co-parenting arrangements? And secondly, how do they manage this parenting arrangement and their relationship with their co-parent? As displayed in Figure 1, two overarching themes (Reproducing the traditional family and Modernising the traditional family) were found to be relevant to both research questions. These seemingly contradictory ideas of both reproducing and modernising the traditional family were prevalent across the sample, highlighting participants’ complex understandings of, and relationships to, the ideal of the ‘traditional family’. With respect to research question one, three themes were identified (Themes 1, 2, and 3) and with respect to research question two, four themes were identified (Themes, A, B, C and D). As depicted in the Figure 1, these themes had complex relationships to the overarching themes of Reproducing/Modernising the traditional family, and this will be explored below. The themes are now outlined in further depth, using illustrative quotations from the data.

### Deciding to become a co-parent

#### Theme 1: Expecting the traditional family: Co-parenting as Plan B

This theme describes the way in which elective co-parenting was not participants’ first choice route to parenthood. Participants reported expecting, and trying to achieve, parenthood within the context of a romantic relationship:

I really wanted a baby and I was like [to my ex-partner] ‘will you just give me one’ and he was like ‘I can’t’ and also there was some problems in that relationship so that ended. (Maya)

Despite having a high desire for parenthood, a number of participants described that their prior relationships had been unsuitable for raising a child and so they sought out other romantic partners with whom they could parent

I came out from a relationship with [my eldest child’s] dad in an awful state … and I had a couple of boyfriends after him and they just seemed to be the same sort of character, and I thought well, I’m really desperate for another child … I wasn’t meeting anybody and that’s when I really started putting some thought into it and going online. (Colleen)

This demonstrates an imagining of parenthood within the context of a romantic relationship, and for many participants, co-parenting felt like ‘the only way I’m going to have a kid’ (Frank). This was due to the lack of available options for single parenthood (‘I certainly didn’t have the money to go through a clinic’ (Colleen)) and the perception that time was running out:

I just wanted to raise a kid and my life has always been about me, and I’m tired of it always being about me, I want it to be about somebody else now. And I didn’t have a girlfriend I wanted to do it with, and I’m getting older, I can’t wait forever, I’m going to do it now. (Louis)

Due to a belief in the traditional family as ideal, some participants found that the decision to pursue parenthood outside of a relationship to be difficult: ‘I didn't know whether this was normal, if it could be done, if anyone else was doing it’ (Arjan). For Colleen, this reimagining of parenthood challenged her traditional values:

I would have liked to have done it within a relationship, you know, what I did goes completely against my values, I’m very old fashioned. I was always no sex before marriage and you have children within a solid relationship, so actually doing this was a huge step for me.

#### Theme 2: Seeking the traditional family by non-traditional means.

Due to a conscious or unconscious belief in the traditional family as ideal, participants saw co-parenting as a way in which to replicate the traditional family, albeit via non-traditional means. This was described by Louis, who also considered surrogacy as a route to parenthood:

Both my parents were married our whole lives, I’m used to that traditional thing, and this was as close as I could get to it in a way. I had no surrogacy history in my family. (Louis)

For a number of parents this was due to a belief that children need a mother and father (‘I looked into insemination but then I thought … I don’t want a child to not have a father’ (Maya)), and that mothers and fathers have different roles:

I want my children to have everything in terms of from their mother. That love, that nurturing, everything, ‘cause these are the kinds of things that a child needs from their mum. (Arjan)

For others it was due to a belief that their child should ‘know where they come from, know what their background … I do think children should have two parents’ (Colleen), and this echoes prior research on LGBTQ + elective co-parenting (Erera & Segal-Engelchin, 2014; Herbrand, 2018a, 2018b). Due to these beliefs, single motherhood was sometimes devalued by participants, who deemed it to be ‘quite selfish … one day those children are going to ask those questions’ (Arjan), or damaging to children:

I had a few friends who were single mums, and their relationships with their children was so intense. And also I was a bit like ‘I don't really want that intensity’ … I hadn't perceived it to be a healthy relationship. (Tanya)

Many mothers in the sample considered, but ultimately rejected, single motherhood. Some fathers also considered single fatherhood, but a number of fathers in the sample approached co-parenting from a different perspective, having been anonymous sperm donors within fertility clinics:

[Sperm donation] just seemed like a no-risk easy thing to do … I don’t have a child right, and I’m 37 so it’s like, how long does it take to enter into a relationship and what’s the likelihood of me getting married and having a kid now, probably not. (Frank)

Co-parenting therefore offered men the opportunity to ‘get to know the person better than you would do if it was just a sperm bank’ (Harry) and have more involvement in their child’s life. As described by Nathan, co-parenting allowed him to ‘actually see my potential kids’. Whilst this may initially seem like the pursuing of modern, involved fatherhood, most fathers pursued parenthood with a primary caregiver mother, suggesting that co-parenting may offer men a route to becoming a father, without the responsibility of ‘involved’ fatherhood:

We did sign an agreement that I forfeit my fatherhood rights, and by that it [would] also happen that there was no financial or legal bindings on me. (Nathan)

More broadly, participants reported pursing parenthood with traditional gendered roles: ‘I had the intention of basically finding someone to be a co-parent with and where the child would live with their mother’ (Arjan). These quotations demonstrate the way in which co-parenting represented a non-traditional route by which participants could seek a family that was, in many ways, traditional.

#### Theme 3: Co-parenting as an improved version of the traditional family

Although co-parenting was often a second-choice route to parenthood, some participants stated that co-parenting offered an opportunity for them to improve upon and modernise the traditional family.

Across the sample, participants were concerned with the fact that ‘one in two marriages will end in divorce’ (Arjan), and participants therefore considered co-parenting to be ‘the most stable relationship’ (Frank):

I think about 80% of families they start to have some personal problems between each other … when you decide to have children like co-parenting, with calm head, with calm heart, you can decide, you can discuss everything. (Sasha)

Participants’ considerations of divorce were often grounded within their personal experiences, and thus co-parenting allowed participants to improve upon their experiences with unstable family relationships.

You want to be like the Disney ideal, even though it’s not realistic. My mum's divorced twice … marriage hasn't been a huge success in my family. (Frank)

For some parents in the sample, co-parenting was envisioned as a modern approach to enabling parenthood outside of a relationship: ‘I wanted another couple of children and also I didn’t want a relationship plus I thought now would be a time to help out a couple of people who didn’t want the relationship either’ (Harry). This was also deemed to offer more freedom in parenting, allowing Louis, for instance to pursue involved fatherhood (‘[co-parenting] does serve that stay-at-home dad thing that I want to do’) and for Maya to pursue equal parenthood:

There’s so much built-in inequality in marriage that co-parenting, it overrides it in a way because it’s not that powerful institution that like psychologically pulls us in like this is my role, this is your role. (Maya)

Co-parenting therefore was chosen by participants as it was perceived to offer the ‘all the benefits of marriage, but none of the drawbacks’ (Arjan), allowing parents to have freedom and control, whilst also enabling them to share the responsibility of parenthood: ‘so that my child had a second parent, and wasn't just reliant on me.’ (Donna)

### Managing the co-parenting arrangement and relationship

#### Theme A: Getting (and staying) on the same page

As described above (see Theme 3), co-parenting was perceived to offer parents an opportunity to ‘modernise’ the traditional family. In practice, this meant that participants prepared extensively for co-parenting, chose their co-parent carefully, and had ongoing, detailed discussions about their co-parenting agreement.

Many participants described thinking carefully about who they wished to parent with (‘We could minus love from the equation, so all we were thinking logically and rationallly’ (Arjan)). Participants reported choosing characteristics that would enable them to be a good co-parent, such as being ‘an honest person and they’ll stay involved’ (Colleen), and a successful parent in general:

I would see if a person [is] mentally and financially is strong enough to have a child … that’s actually the first focus, and the second focus is the distance. (Nathan)

Participants’ arrangements varied substantially, from little expectation of father involvement (‘whoever the father was I’d want them to be involved, even if it was just a letter or a picture once a year’ (Colleen)) to co-parents living in the same house or town:

We're pretty even stevens … Once breastfeeding got established, [child] would spend 4 nights a week with me in the room, and then I would express, and then he would spend 3 nights in the week with [co-parent]. (Tanya)

Regardless of the arrangement, parents spoke about the importance of being on the same page:

The more that we talked about it, the more that I thought well actually this could work, it's us and we get on and it's different, yes and there's no real blueprint for it, but it could work, because we were both, we have very similar value systems (Tanya)

This represents a modern approach to family, in that participants chose their co-parent based on similar values and parenting characteristics. A number of participants drew up contracts, despite them not being legally binding:

Once I was pregnant he did get a contract from his lawyer but he said they said ‘this doesn’t hold up in court’ … it was more just a conversational starter but we did actually write it up and sign it and get it notarised and all that (Maya)

However, co-parents were not always on the same page in terms of their agreement:

I said at least we will make contract, I will make all preparation, and I will send to you. He said … [it’s] better to have some communication based on trust … But now I think that was my mistake. (Sasha)

Notably, a number of participants highlighted that ‘despite all the best preparation and planning in the world, you never know what's gonna happen’ (Arjan). Indeed, Maya reported that, despite extensive discussions and planning, ‘I probably should’ve waited til we had a disagreement to see how it [laughs], how he responded but no it was, kind of perfect, too perfect’.

Participants therefore noted the importance of ongoing discussions about their co-parenting arrangement and relationship, which was found to be difficult to manage alongside the demands of parenting:

[You need] as much discussion as possible about who's going to have the child, and what your different roles are … that's where we've been falling down because, I'm just so busy, or so tired, or something, and can't be- It's the last thing I want to do, you know? (Donna)

#### Theme B: ‘We’ve got that mutual respect and understanding’: friendship as vital

As discussed in Theme A, participants often gave a lot of thought to planning/managing their parenting arrangement, but they also ascribed a high level of importance to the affective side of their co-parenting relationship. A number of participants characterized themselves and their co-parent as ‘two friends that had a baby’ (Maya) and this friendship was seen to facilitate a successful co-parenting relationship:

Having friendship and mutual respect, that's something that we really value and [respect] cause we both, me and [co-parent] never had that during our previous marriages. (Arjan)

In this way, participants experienced co-parenting as a way to improve upon their past relationships and modernise the traditional family. A good relationship between co-parents was thought be particularly important due to the ‘life changing commitment’ (Arjan) of raising a child:

Don't just do [co-parenting] to have a child, because actually, a child binds you together forever, right? And if you don't want to be bound together forever with someone, then you shouldn't have a child with that person. (Tanya)

Indeed, Maya reported not considering the ongoing nature of the co-parenting relationship enough when choosing who to conceive with:

You don’t want someone who just wants to be a good father, you want someone who’s gonna be a good partner to you … they have to be willing to love you and care for you as well, and I never realised that that was gonna matter but it was a pretty big deal.

Some participants who had been friends prior to co-parenting found that this was an advantage when starting the journey to parenthood:

The fact that we've been friends for such a long time, the fact that we are really close, that's made a massive difference. (Tanya)

However, others found that a pre-existing relationship was not necessarily a predictor of success:

When our relationship changed a lot, and we lost a lot of our friendship, is when [child] was a baby because it was just so new to me and just stressful, and he would suggest things and I just thought they were things I'd already tried. (Donna).

This highlights that when practices associated with friendship (such as effective social support) were lacking, co-parenting relationships became strained. Building a co-parenting relationship was found to be challenging for participants, as ‘you do have to learn to trust someone at a level that you wouldn't necessarily’ (Tanya). Some participants reported that their trust in their co-parent was damaged, as they failed to engage in basic parenting practices:

After [child] was born, we have a very, not very bad, but not good connection … he doesn't ask about [child] or anything … I sent him photo and some videos but he sent me emoji. He didn't see her on Skype. (Sasha)

Louis described hoping that the lack of a romantic relationship would enable a successful co-parenting arrangement: ‘All my ideas I had was well this is great because … we don’t have that personal history to get in the way and make it get horrible’. He then described his unpreparedness for relationship difficulties with his co-parent (‘how could you be so angry at me, you don’t even know me’), demonstrating that previous suggestions that elective co-parenting are easier to navigate due to a lack of previous conflict (Segal-Engelchin et al., 2005) may be incorrect.

#### Theme C: ‘She makes [the decisions], I stick by them’: Taking on gendered roles

Across the sample, most participants’ parenting arrangements followed traditional gendered norms. Generally, mothers were responsible for being their child’s primary caregiver, and the father played a secondary role as the mother’s ‘helper’:

[Child] is my son, my son definitely, but [co-parent] comes round usually on Saturdays for a few hours … I can phone him up and [say] can you come and babysit and he will. (Colleen)

Mothers spoke about engaging in practices of maternal gatekeeping, and wanting to control their co-parent’s involvement and contact:

Him being in [a different country] … that kind of protected me in a way. The children would be here with me, and he would have as much involvement as I let him, basically … and that suits me very well. (Donna)

Relatedly, Colleen described initially being reluctant to include her co-parent on her child’s birth certificate, due to fears that he would have power to take her children away, but wanting to do so ‘for my son’s sake, because I don’t want him to get older and go well why is my dad not on my birth certificate’. This highlights the importance of documentation as an act of family display (Zadeh et al., 2022), as it enabled participants to display to their child that they had a ‘proper’ family, according to hegemonic ideals.

Some fathers reported being satisfied with arrangements controlled by mothers. For instance, Harry who had entered into co-parenting arrangements with two separate women, said ‘I’m happy with it yeah … I’ve seen [my child] now six times in the last five years, so it’s as and when mum lets me’. In these cases, fathers can be seen to be making a trade-off between parental rights and responsibilities. Indeed, fathers who were less involved described that the mother was responsible for everyday parenting decisions, related to child discipline, for example: ‘I just let the mum decide. She’s doing all the work’. (Frank).

For some fathers, this lack of control was difficult, as described by Frank when asked how satisfied he was with his arrangement:

Oh well it’s better than nothing … I would have liked to live in the same city and then I could see her at the weekends obviously a lot easier … But maybe that’s an idealistic thing, maybe her mum doesn’t want that, right, maybe she just wants me to see her once a month.

Given the lack of available parenthood options for single men (Jones et al., 2022) it is possible to suggest that men enter into non-ideal co-parenting arrangements due to a perceived lack of options: ‘I think I just wanted to have a kid so bad I kind of jumped in’ (Louis).

Participants also spoke about their parental roles in gendered ways. For instance, Arjan described his and his co-parent’s roles in traditional gendered terms: ‘She's a full-time mother, home-maker … and I contribute as much as I financially can every month’. Harry described himself as a ‘father figure sort of thing … I think [child] finds [me] a bit more fun to be around and I’m not one for rules’. Colleen also described her child’s father as doing ‘normal dad stuff’, demonstrating that gendered family practices that reproduce the traditional family may enable co-parents to display their family as a ‘normal’ family. On this point, Donna described feeling similar to other mothers at school because ‘my complaints about [co-parent], they're actually very similar to my friends’ complaints about their husbands’ and this highlights that even negative instances of family display (e.g. complaining about a co-parent) may enable co-parents to display themselves as a ‘standard’ family to others.

#### Theme D: ‘I just say it’s complicated’: Defining the relationship and managing blurred boundaries

Participants reported that, in the absence of a defined relationship with their co-parent, it could be difficult to define and manage the boundaries of their relationship. Participants’ relationship to the idea of the ‘traditional family’ was influential in their management of their arrangement.

Participants generally found that describing their family to others could be difficult: ‘[people] don't even know what co-parent is’ (Arjan). Some participants therefore spoke about their family in vague terms (‘I just say it’s complicated’ (Colleen)), described their relationship as married parents might (‘I would just say ‘my partner and I'’ (Tanya)) or relied on societal understandings of co-parents as separated parents:

When I go across customs I’ll say I’m going to see my daughter, and they’re like where is she? Well she lives with her mum, and I just leave it at that, people will assume we broke up or something. (Frank).

This demonstrates that successful family display is particularly important in certain situations, and highlights the unique challenges facing international co-parents.

All participants were heterosexual (although their co-parents’ sexual orientations varied) and a number of participants reported that either they, or their co-parent, had developed romantic feelings for the other:

She had kinda said although she was very happy with the arrangement, everything was fantastic, she had developed feelings for me. So rather than just being friends, like co-parents, liking me in a romantic sense, and asking to live together and stuff, and be – I hate this word you know – a traditional family. (Arjan)

Arjan also reported an ongoing and exclusive sexual relationship with his co-parent, which he defined as ‘[not] a romantic relationship. I think I’m pretty firm about that … It’s just co-parents with benefits maybe’. Therefore, despite having, in many ways, a ‘traditional’ family (see also Theme C, taking on gendered roles), some participants expressed discomfort with being labelled as such, demonstrating a discrepancy between family display (as a non-traditional family) and family practices (as a traditional family). Importantly, Arjan viewed avoiding the ‘traditional family’ as avoiding divorce: ‘what's the natural progression – it's gonna be divorce. It's not gonna be good for us, it's certainly not gonna be good for our kids’, suggesting that discrepancies between family practices and family display may be explained by family thinking (i.e. a rejection of the ideal of romantic relationships lasting forever).

In contrast, Frank reported desiring to be in a traditional family, due to ideals about what ‘proper’ families should look like:

I should be in love with [co-parent] and come home and then kiss her on the cheek and then eat our dinner together, so I want that sometimes, but I use reason to think, well … if I was with her maybe I would cheat on her, maybe something would happen and it would be an unhappy relationship. (Frank)

This demonstrates that the traditional family is both idealized (evidenced in Frank’s understanding of how he ‘should’ be with his co-parent) and devalued (evidenced in Frank’s assumption that he would be the one to cheat on her), which ultimately serves to reproduce the married traditonal family as an unattainable ideal.

Tanya (who was co-parenting with a gay man) reported navigating dating and romantic boundaries:

I went on one date. And I didn't tell [coparent] to begin with, because I wasn't sure whether I wanted to do it or not. But anyway, when I did tell [co-parent], [co-parent] was quite upset, because he felt betrayed and felt as if it destabilised our whole situation.

Maya highlighted that conception via intercourse had complicated boundary management with her co-parent:

I had [co-parent] acting very, not like a co-parent and more like a boyfriend and I had to draw a boundary and be like ‘look we’re friends doing this, I know we had sex to make the baby but I think I was very clear’ and he was very upset by that … that made me angry, scared.

These demonstrate that negotiating and asserting intimacy boundaries may be particularly challenging in co-parenting arrangements, given the lack of a cultural script for what these relationships involve.

## Discussion

Within this study, we have explored two research questions: why do people decide to enter into elective co-parenting arrangements? And how do they manage this parenting arrangement and their relationship with their co-parent? The findings demonstrate that participants tended to idealize the traditional family and view co-parenting as a ‘second choice’, confirming the findings from previous research (Jadva et al., 2015; Segal-Engelchin et al., 2012). Deciding to co-parent could be challenging, as participants renegotiated their ideas of what families looked like, but also liberating, in allowing them to fulfil their parenting desires and ideals outside of a romantic relationship. Despite substantive changes in parenthood ideology, notions of the ‘traditional family’ appeared to remain prominent. Whilst participants’ co-parenting arrangements varied considerably, they were largely similar in that they tended to follow gendered patterns, thus extending our previous knowledge of cis LGBQ + co-parents to cishet parents (Dempsey, 2012; Erera & Segal-Engelchin, 2014; Gahan, 2019; Herbrand, 2018a, 2018b). Mothers tended to be the primary caregivers, and sometimes engaged in maternal gatekeeping, suggesting that ideologies of intensive motherhood may be prominent amongst cishet elective co-parents. This extends our knowledge of how societal expectations around parenthood influence the lives and experiences of parents in diverse family forms.

Participants planned out their co-parenting arrangements carefully, often drawing up contracts and negotiating with their co-parent, demonstrating that elective co-parenting represents a novel way in which to pursue modern parenthood. Additionally, co-parents aimed to manage their co-parenting relationship with friendship, trust, and shared values. Some participants appeared to be successful in realizing this aspiration, and this study highlights that co-parenting with a friend or acquaintance can be a fulfilling and positive experience. However, in some cases, relationship management could be challenging, as participants struggled to define their relationship and navigated differing expectations with their co-parent. Research on other family types has found that pre-parenthood expectations of couple parenting equality are not always met (e.g. Faircloth, 2021; Shaw et al., 2022), and this study suggests that such violations of equality expectations can persist when parenting with a friend or acquaintance.

### Grappling with tradition

Nordqvist (2017, p. 878) suggests that we need to ‘develop a sociological gaze more sensitive to the relationship between activities and the feelings, imaginations, dreams or claims with which they are entwined’. The current study has developed this perspective by analyzing data on the experiences of elective co-parents, a group that is perhaps particularly worthy of study when considering parenting practices and ideals, given the lack of other roles that participants shared (e.g. a current or past romantic relationship). Across the sample, participants both reproduced and modernized their ideal of the traditional family. This echoes research on solo mothers, which has found that mothers realign their family ideals (of pursuing parenthood with a partner) with their family realities (of pursuing parenthood without a partner) (Graham, 2018). Individuals who pursue parenthood in non-traditional ways have been conceptualized as ‘moral pioneers’ (Graham, 2018; Rapp, 1988), as they aim to align their route to parenthood with dominant notions of ‘good’ parenthood. This can also be seen within the current sample; parents who were not able to fulfil their ideal of parenting within a romantic relationship, leaned into other dominant notions of ‘good’ parenthood (i.e. involving a biologically related mother and father, and involving intensive mothering practices).

Whilst a minority of parents pursued co-parenting arrangements with highly involved fathers, participants’ arrangements were often highly gendered, with mothers being the primary caregiver and largely responsible for parental decision making. Research with mothers who are co-parenting post-separation has found that being a ‘part-time parent’ is challenging to the ideals of intensive motherhood (Markham & Coleman, 2020), suggesting that electiveco-parenting arrangements allowed mothers to fulfil these ideals. Within our sample, motherhood responsibility was therefore deemed obligatory, whereas fatherhood involvement was often deemed optional, echoing research with gay men who pursue parenthood with lesbian couples (Dempsey, 2012). Previous research on elective co-parents has highlighted that women are more likely to have planned their parenting arrangement than men (Jadva et al., 2015), and the current study’s findings add nuance to this research, suggesting that men may lack power in co-parenting arrangements, as instances of maternal gatekeeping were common. For some fathers, their lack of control was a trade-off between rights and responsibilities that they were happy to make, but for others this could be difficult. Given the stigma directed towards single male parents more generally (Jones et al., 2022), more research is needed on the experiences of fathers who wish to be highly involved parents.

Paternal involvement has been characterised by three components: interaction, availability, and responsibility (Pleck, 2010a); fathers in the current sample were more involved in terms of interaction and availability, rather than responsibility. Whilst fatherhood responsibility was therefore, in some cases, deemed optional, the existence of a father was deemed crucial for the child, and here we can see a discrepancy between family display and family practices. A number of mothers (and some fathers) seemed to view the father’s role as little beyond that of a symbolic presence, a father *figure* rather than a father, which ties into traditional notions of fathers as moral role models, signifying their key role in the socialization of healthy children, but with a limited role in day-to-day family practices (Freeman, 2003). Participants wanted their children to know and have a father (due to family discourses that idealise two-parent families and the knowledge of genetic origins) but wanted to retain control over parenting decisions (due to intensive mothering ideologies), and as such engaged in maternal gatekeeping to limit the father’s involvement and contact (Fagan & Barnett, 2003).

Within the current sample, there were often discrepancies between family display and family practices. For instance, in terms of viewing fatherhood as a symbolic presence, family display (i.e. displaying that their child has a father) was different to family practices (i.e. not having an involved father), demonstrating that contradictory ideals of new and traditional fatherhood are still highly relevant. Such findings support Nordqvist’s (2017) suggestion that family scholars need to engage with the relationship between family practice and family discourse. Participants’ adherence to gendered patterns of family life may also be viewed as an attempt to engage in successful family display, and to highlight the similarities between themselves and the idealised traditional family. Moreover, although some participants expressed discomfort with being seen as a ‘traditional family’, this was generally grounded in their personal/family history of divorce. Therefore, the refusal to display as a ‘traditional family’, despite being traditional in family practices, in some ways reinforces the traditional family as an unattainable ideal.

### A ‘pure’ parenting partnership?

The seemingly contradictory ideas of modernising and reproducing the traditional family can be further explained by theorisations of intimacy. Sociological scholarship has focussed on the modern pursuit of a ‘pure relationship’, involving equality, mutual trust and respect (Beck & Beck-Gernsheim, 1997; Giddens, 1992), and indeed the perils of this pursuit, in that such relationships are still structured by inequalities (Jamieson, 1999)**.** Whilst participants in the current study were aware of the limitations of idealizing romantic relationships, in some cases participants pursued the ‘pure parenting partnership’, in that they idealised co-parenting arrangements, assuming that they would be able to be managed with shared values, trust and respect. Whilst some participants did manage their relationships in this way, others reported that their arrangement was difficult to manage in practice, and all parenting arrangements were structured by gender norms and inequalities (Jamieson, 1999).

Giddens (1992) suggests that kinship relations have transformed over time, and whilst previously trust and commitment amongst kin was a given, the rules and nature of kinship now have to be negotiated. Negotiation of commitment amongst co-parents is a particularly important practice, given that co-parents are not kin, but must engage in activities typically practiced with kin. Some participants reported carefully negotiating their co-parenting arrangement, drawing up contracts to outline their rights and responsibilities as parents. This demonstrates the importance of ‘documenting family’ as an aspect of family display, and whilst Zadeh et al.’s (2022) article covered instances of official documentation, in the current study, participants drew up co-parenting agreements that were not legally supported, demonstrating that ‘documenting family’ is an important discursive display practice. Practically, the findings suggest that co-parents may benefit from the support of mediators or lawyers with specific knowledge of co-parenting arrangements, to enable them to most effectively draw up satisfactory arrangements.

It has been noted that it is highly important to investigate the outcomes of co-parenting arrangements that have been made online (Jadva et al., 2015; Ravelingien et al., 2016), as these arrangements may be less sustainable. Within this study, similarities were seen across co-parenting relationships formed both online and offline. The findings instead suggested that there were qualitative differences in the way that successful and unsuccessful co-parenting relationships (as defined by participants) were managed. Drawing upon Feinberg's (2003) four key components of co-parenting relationships: (dis)agreement over childrearing, satisfaction with the division of labour, support/undermining of the parental role, and the joint management of family interactions, all of these aspects were found to be important to participants’ relationship management. However, one key aspect of successful co-parenting not captured by Feinberg’s (2003) model is the ongoing practice of (re)defining of the relationship, and managing blurred boundaries. Of particular note within the current study is the complex negotiation of boundaries of intimacy, including sexual, romantic and emotional boundaries, that participants undertook. This negotiation, unreported in prior research on elective co-parenting, may be particularly important in elective co-parenting arrangements, given the lack of a cultural understanding of what co-parenting relationships and arrangements involve.

Couple relationship satisfaction has been found to be an important factor for child psychological adjustment (Nicolaus et al., 2021) and it is therefore important to investigate child outcomes within elective co-parenting families. Forthcoming work, including the LGBTQ + co-parents who also took part in this study, will highlight family functioning and parent–child wellbeing within these arrangements (Foley et al., forthcoming). Future research also needs to explore children’s thoughts and feelings about being in a co-parenting family, in order to understand whether parents’ beliefs about what their child needs and wants aligns with their children’s views.

### Future of ‘family’ theories?

This study took a unique theoretical approach to understanding the motivations and experiences of elective co-parents, utilizing sociological theorisations of family practices, family display and family thinking. The findings highlight the benefit of using these theorisations in tandem, as participants’ family practices, family display and family thinking influenced each other in a myriad of ways.

Throughout this article we have utilised the labels of the theory as outlined by the original theorists (family practice/display/thinking). It is important to note that participants did not necessarily define their co-parent as a family member, and it can thus be questioned whether parents were engaging in acts of *family* display. For instance, engaging in gatekeeping and boundary keeping practices were important in reinforcing and redefining the co-parenting relationship as one that was not a marriage or family relationship. Moreover, whilst the majority of participants saw their co-parent as a friend, they saw their co-parent’s most important role as their child’s parent and family. Participants therefore engaged in family display towards their child and others that they were a ‘proper’ family, as outlined by dominant ideals that de-legitimise fatherless families (Freeman, 2003). These findings add nuance to our understanding of family practices and display, and demonstrate that theorists need to be attuned to complex definitions and displays in diverse family forms.

### Strengths and limitations

The current study provides a novel insight of the experiences of elective co-parents, and is the first study to qualitatively explore the experiences of cishet mothers and fathers. The study’s strengths include the unique theoretical approach and the inclusion of both mothers and fathers, which enabled an in-depth exploration of gendered parenting practices. The study’s broad inclusion criteria (i.e. anyone who identified as a co-parent) meant that a wide diversity of experiences were captured, but also meant that participants had vastly different experiences. Future research could focus specifically on, for example, the experiences of co-parents who live together, or the experiences of co-parents who co-parent internationally. Moreover, the international sample meant that we were not able to pay particular attention to the unique legal and cultural environments that shaped participants’ experiences.

## Conclusion

This study of elective co-parents allows for the de-coupling of co-parenting relationships from romantic relationships. The findings demonstrated that, in the absence of a romantic relationship, participants experienced a tension between reproducing and modernising the traditional family. Although co-parenting was generally a second-choice route to parenthood, participants aimed to approach co-parenting in a considered manner, choosing co-parents based upon shared values and managing their relationships with trust and friendship. Some participants imagined and managed their parenting practices in ways which transcended traditional expectations of family life. However, gendered parenting roles were found to be common across the sample and participants’ understanding and negotiations of their co-parenting arrangements were complex. Individuals undertaking elective co-parenting arrangements can therefore be seen as ‘moral pioneers’ (Rapp, 1988), as they are at the forefront of renegotiating family values and ideals outside the context of romantic relationships. The findings highlight the pervasiveness of traditional parenthood ideologies, and demonstrate that parents in diverse family forms grapple with tradition, as they endeavour to imagine and pursue parenthood on their own terms.

## Note

1 We use the term ‘elective co-parenting’ (Jadva et al., 2015) or ‘co-parenting’ within this article to refer to an arrangement in which two (or more) parents who are not in a romantic relationship decide to conceive a child together, with both parents involved in the child’s life to some degree. Where other types of co-parenting are referred to (such as co-parenting post-separation), this is made clear.
